# Modulating the host immune response to fight against COVID-19: Where are we in 2021?

**DOI:** 10.1080/21505594.2021.1943275

**Published:** 2021-07-05

**Authors:** Decsa Medika Hertanto, Henry Sutanto, Bayu Satria Wiratama, Citrawati Dyah Kencono Wungu

**Affiliations:** Department of Internal Medicine, Faculty of Medicine, Airlangga University, Surabaya, Indonesia; Department of Internal Medicine, Soetomo General Hospital, Surabaya, Indonesia; Department of Cardiology, CARIM School for Cardiovascular Diseases, Maastricht University, Maastricht, The Netherlands; Department of Epidemiology, Biostatistics, and Population Health, Faculty of Medicine, Public Health and Nursing, Universitas Gadjah Mada, Yogyakarta, Indonesia; Graduate Institute of Injury Prevention and Control, College of Public Health, Taipei Medical University, Taipei, Taiwan; Department of Physiology and Medical Biochemistry, Faculty of Medicine, Airlangga University, Surabaya, Indonesia; Institute of Tropical Disease, Airlangga University, Surabaya, Indonesia

**Keywords:** COVID-19, coronavirus, SARS-CoV-2, immunomodulation, immunology, immune system, pharmacotherapy, drug repurposing

At present, while some countries are dealing with the continuously increasing incidence, some of the others are currently facing the 2019 coronavirus disease (COVID-19) resurgence, entering the second and third waves of the pandemic. Several determining factors could be attributed to such global phenomenon, including the emergence of novel severe acute respiratory syndrome-associated coronavirus type-2 (SARS-CoV-2) pathogenic variants (e.g. N501Y, E484K, B117 and N440K) and the nonexistence of effective pharmacological modalities for COVID-19. Despite the remarkable success of SARS-CoV-2 vaccines development, the treatment modalities for COVID-19 remains limited. Several repurposed drugs that were deemed to be useful were ineffective and even harmful [1,2]. Of those are the antimalarial drug chloroquine, antimicrobial azithromycin and protease inhibitors ritonavir/lopinavir, which were no longer recommended because of their potential cardiotoxicity [2,3]. Meanwhile, the efficacy of other repurposed drugs (e.g. ivermectin and favipiravir) are being disputed due to conflicting results among studies [4–7]. To date, only antiviral remdesivir promoted significant clinical improvement and therefore, is authorized by major drug safety regulators for COVID-19 [8]. Meanwhile, although the pathophysiology of COVID-19 has not been fully elucidated, several evidences pointed toward the strong involvement of proinflammatory cytokines in the genesis of systemic hyperinflammation, multiorgan dysfunction and death [9]. Such observations provided a premise that immunosuppression could potentially be beneficial in COVID-19. However, higher comorbidities, increased rate of intensive care and in-hospital mortality were observed in immunocompromised COVID-19 patients, indicating the potential risk of immunosuppression in COVID-19 [10]. Therefore, we aim to provide a current update on the documented effects of immunosuppressive medications (e.g. corticosteroids, interleukin (IL) and kinase inhibitors) and immunomodulators (e.g. interferons, non-SARS-CoV-2 specific immunoglobulin and convalescent plasma) in COVID-19 management and propose some potential immunologic targets (see Figure 1(a)).

**Figure 1.**
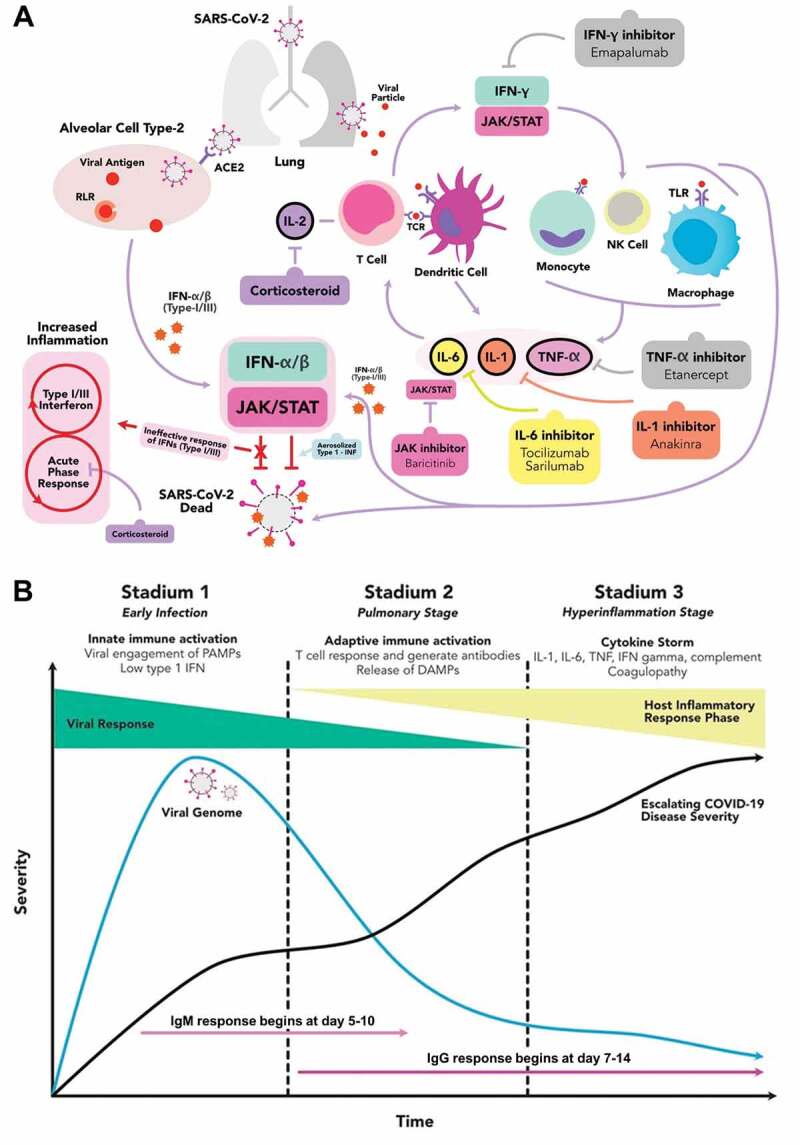
The hyperactive immune response in SARS-CoV-2 infection, druggable immunologic targets and the stages of severity in COVID-19. (a) SARS-CoV-2 enters the infected person via the respiratory tract and attaches to the ACE2 receptors in type-2 alveolar cells of the lungs. It subsequently activates the retinoic acid inducible gene-(RIG) I-like receptors (RLRs), an initiator of antiviral immune responses activation. Together with the intrinsic response to the viral particles, they induce hyperactive inflammatory response, marked by the activation of proinflammatory cytokines-releasing cells. Several immunologic targets were identified to have an important role in the COVID-19-mediated cytokine release syndrome/cytokine storm. Thus, some pharmacological agents are employed to alter those targets, prevent the viral entry and replications, and reduce the COVID-19-induced hyperinflammation. (b) The COVID-19 can be divided into 3 stadiums: the early infection, the pulmonary and the hyperinflammation stages. In the early infection, the viral load starts to increase and at some points, it begins to activate the host immune response. While the disease progresses into a more severe state, the proinflammatory cytokines build up and start to form antibody against the virus. When the disease is not promptly treated, COVID-19 may fall into the hyperinflammation stage, multiorgan failure and death. (ACE2 = angiotensin converting enzyme type-2; CoV = coronavirus; DAMP = damage-associated molecular pattern; IFN = interferon; IL = interleukin; JAK = janus kinase; NK = natural killer; PAMP = pathogen-associated molecular pattern; SARS = severe acute respiratory syndrome; STAT = signal transducer and activator of transcription; TCR = T-cell receptor; TLR = toll-like receptor; TNF = tumor necrosis factor)

Hyperactivation of host immune system is a hallmark of COVID-19 severity. Ample evidences have documented an elevated leukocyte count and increased inflammatory mediators, such as C-reactive protein (CRP), procalcitonin, proinflammatory cytokines (e.g. IL families) and chemokines (e.g. CCL and CXCL families), in moderate to severe COVID-19 patients. Such hyperinflammatory response initiates cytokine storm and may contribute to the uncontrolled apoptosis, vascular leakage, thromboembolism, multiorgan damage and death [9]. In COVID-19, ***corticosteroid*** in its inhaled form was employed to alter the SARS-CoV-2 replication-transcription complex and inhibit the viral ribonucleic acid (RNA) replication [11]. Corticosteroid also lowered the COVID-19-associated mortality in patients with acute respiratory distress syndrome and reduced the need for oxygen supplementation [12]. Similarly, another study showed that corticosteroid lowered the 28-day mortality in COVID-19 patients with mechanical ventilation or oxygen supplementation. Interestingly, the benefit was not attained in patients without any means of respiratory support [13]. Moreover, in adults with non-severe COVID-19, corticosteroid was even associated with worse clinical outcomes, including a prolonged hospital stay and a higher risk of disease progression [12], highlighting the potential benefits of corticosteroids in moderate to severe COVID-19 patients.

Several interleukins are held responsible for COVID-19-mediated cytokine storm (e.g. IL-1β, IL-6 and IL-18), therefore ***interleukin inhibitors*** could be advantageous. A previous study showed that anakinra, an IL-1 inhibitor, significantly lowered mortality in patients with COVID-19-induced hyperinflammation and respiratory failure, while IL-6 inhibitors (e.g. tocilizumab and sarilumab) were effective solely in patients with high CRP or low lactate dehydrogenase [14]. Moreover, IL-6 inhibition also improved survivals in severe COVID-19 patients receiving intensive organ support [15] and was consistently associated with a lower risk of death [16], underlining the prospective benefits of interleukin inhibition in COVID-19. Meanwhile, the contribution of numerous kinases (e.g. ABL, NAK, CDK, PI3K/AKT/mTOR, ERK/MAPK and JAK) was also observed in COVID-19, opening a path for ***kinase inhibitors*** in COVID-19 management. Kinases play important roles in viral entry, replication and life cycle, intracellular membrane trafficking and possess an immunomodulatory effect that could be useful against COVID-19-mediated hyperactive immunity. Indeed, baricitinib, a janus kinase (JAK) inhibitor, inhibited major protein phosphorylation, altering the signal transduction that initiates host immune response and inflammation [17]. The combination of baricitinib and remdesivir promoted faster recovery and accelerated clinical improvement than remdesivir alone. Moreover, it was associated with fewer serious adverse events in patients with noninvasive ventilation or high-flow oxygen support [18]. However, a negative result was reported with imatinib, an ABL inhibitor, which was shown not to inhibit SARS-CoV-2 entry and replication in an *in-vitro* study [19], indicating the prominence of several kinases among others in COVID-19 pathogenesis.

In addition to immunosuppression, immunomodulators are expected to restore the immunologic homeostasis in COVID-19. Conventionally, in the presence of viral pathogens, the host cells produce and release cytokines-derived ***interferons (IFNs)*** as a self-defense mechanism. However, SARS-CoV-2 could release molecular (anti-IFN) defenses to evade host innate immunity at the early stage of infection, diminishing the effect of intrinsic IFNs in limiting viral replication and spreading [20]. Indeed, nebulized IFNα-2b speeded up the clearance of SARS-CoV-2 from the respiratory tract and yielded a reduction in systemic inflammation [21]. Additionally, the combination of IFNβ-1a with antimalarial drug and/or protease inhibitors promoted discharge at day-14 and lowered the 28-day mortality [22]. Nonetheless, the suppression of intrinsic IFN by SARS-CoV-2 is only present at the early phase of the disease, while in the later stages, the type-I IFN response remains robust [23]. Thus, despite these promising results, more data investigating the efficacy of IFNs in various COVID-19 stages are required.

Donors’ plasma may provide passive immunity against SARS-CoV-2 infection. ***Non-SARS-CoV-2 specific intravenous immunoglobulin or IVIg*** is mass-produced from pooled plasma of donors and employed to fight against nonspecific pathogens. In COVID-19, although the exact mechanism is unclear, it is hypothesized to modulate inflammation (via the presence of anti-idiotypic antibodies and IgG dimers blocking the FcγR activation on innate immune effector cells), promote complement scavenging and alter the regulation of T lymphocytes (e.g. Th1 and Th17) [24]. Additionally, IVIg also decreased plasma IL-6 and CRP levels [25]. The administration of IVIg in severe COVID-19 patients who did not respond to the original treatments improved clinical outcome and reduced mortality [26]. Additionally, an early IVIg therapy reduced hyperinflammatory response, hospital stay, 28- and 60-day mortality, improving multiorgan physiology and clinical outcome of severe COVID-19 patients [25], which effects are more prominent in patients with no comorbidities or who are treated at an earlier stage [27]. On the other hand, ***hyperimmune globulin and convalescent plasma*** are obtained from previously infected people with high antibody titers against specific pathogens (e.g. SARS-CoV-2). A retrospective case-control study reported that convalescent plasma reduced the oxygen demands at day-14 post-transfusion and improved survivals in severe and critically-ill COVID-19 patients [28]. However, it was opposed by randomized control trials (RCTs) which reported no benefit in all-cause mortality or other clinical outcomes compared with placebo or standard care [29]. Because the RCTs also included moderate to severe COVID-19 patients, this disagreement might not be due to the disease severity.

Overall, the majority of previous clinical trials indicated that both immunosuppression and immunomodulation were effective in severe COVID-19 conditions requiring respiratory support and ventilation, while in non-severe disease phase, immunologic treatments might elicit worse outcome (e.g. corticosteroid) or no significant improvements of clinical outcome or mortality. This fits well with the identified disease pathophysiology (see Figure 1(b)) implying the hyperinflammatory state with cytokine release syndrome in severe COVID-19. In such conditions, inhibition of proinflammatory cytokines with immunosuppressive agents could reduce the damaging consequences of rogue inflammation and immunomodulation might restore the host immune regulation. On the other hand, in the early phase, immunologic treatments could disrupt the activation of immune response against viruses and therefore, could be harmful.

In the future, several novel immunologic targets such as tumor necrosis factor (TNF)-α inhibitors, complement inhibitors, RLR and mTOR inhibitors, NLRP3 inflammasome inhibitors, TLR modulators, IL-18 inhibitors and possibly mesenchymal stem cell secretome could be tested due to their reported significance in COVID-19 pathogenesis. TNF-α inhibition has been shown to reduce mortality and hospital admission for COVID-19 [30], although further clinical study on the efficacy of TNF-α inhibitors in COVID-19 patients without immune-related comorbidities is warranted. Meanwhile, a preliminary data demonstrated the presence of complement hyperactivation in COVID-19 [31]. Therefore, several clinical trials have been started to investigate the potential role of complement inhibitors in COVID-19 [32], e.g. the combination of C5 inhibitor ravulizumab and JAK inhibitor baricitinib, which is now entering the phase IV clinical trial [33]. Next, retinoic acid inducible gene-(RIG) I-like receptors (RLRs) are activated in the presence of viral RNAs and initiate the production of inflammatory mediators, including type-I and III IFNs (see Figure 1(a)) [34]. Therefore, the inhibition of RLR is expected to alter the host-virus interactions and prevent the activation of excessive inflammatory response. The activation of NLRP3 inflammasome in COVID-19 facilitated the initiation of major proinflammatory cytokines, such as IL-1β and IL-18 [35]. Therefore, inhibitions of NLRP3 inflammasome and its downstream mediators could minimize the hyperinflammatory state. Lastly, although is still early, the possible contributions of mesenchymal stem cells and their secretome in COVID-19 management are being investigated [36]. At present, aforementioned potential targets remain at the investigational stage and further extensive experimental and clinical studies are required to evaluate their efficacy in COVID-19.

## Data Availability

Data sharing not applicable – no new data generated.
